# Differential patterns of definitive host use by two fish acanthocephalans occurring in sympatry: *Pomphorhynchus laevis* and *Pomphorhynchus tereticollis*

**DOI:** 10.1016/j.ijppaw.2019.01.007

**Published:** 2019-02-01

**Authors:** Marie-Jeanne Perrot-Minnot, Emilie Guyonnet, Loïc Bollache, Clément Lagrue

**Affiliations:** a Biogéosciences, UMR 6282 CNRS, Université Bourgogne Franche-Comté, 6 Boulevard Gabriel, 21000, Dijon, France; b Chrono-environnement, UMR 6249 CNRS, Université Bourgogne Franche-Comté, 16 Route de Gray, 25000, Besançon, France; c Department of Biological Sciences, University of Alberta, Edmonton, Alberta, T6G 2E9, Canada

**Keywords:** Compatibility, Complex life-cycle, Freshwater, Host range, Index, Reproduction, Specificity

## Abstract

Parasites with complex life-cycles and trophic transmission are expected to show low specificity towards final hosts. However, testing this hypothesis may be hampered by low taxonomic resolution, particularly in helminths. We investigated this issue using two intestinal fish parasites with similar life-cycles and occurring in sympatry, *Pomphorhynchus laevis* and *Pomphorhynchus tereticollis* (Acanthocephala). We used species-specific ITS1 length polymorphism to discriminate parasite species from 910 adult acanthocephalans collected in 174 individual hosts from 12 fish species. Both *P. laevis* and *P. tereticollis* exhibited restricted host range within the community of available fish host species, and transmission bias compared to their relative abundance in intermediate hosts. The two parasites also exhibited low niche overlap, primarily due to their contrasting use of bentho-pelagic (*P. laevis*) and benthic (*P. tereticollis*) fish. Furthermore, parasite prevalence in intermediate hosts appeared to increase with taxonomic specificity in definitive host use. Comparison of *P. laevis* and *P. tereticollis* adult size in the two main definitive hosts, barbel and chub, suggested lower compatibility towards the fish species with the lowest parasite abundance, in particular in *P. laevis*. The determinants of low niche overlap between these two sympatric acanthocephalan species, and the contribution of definitive host range diversity to parasite transmission success, are discussed.

## Highlights

• The fish acanthocephalans *P. laevis* and *P. tereticollis* show moderate specificity and low niche overlap.
• Transmission bias from shared intermediate hosts towards either benthic or bentho-pelagic fish is evidenced.
• Decreased taxonomic specificity towards fish hosts matches with lower prevalence in intermediate hosts.
• Lower worm size in the host with the lowest abundance calls for further investigation of compatibility filter.
• Low taxonomic resolution within some parasite species complex hinders accurate estimate of host use pattern.

## Graphical abstract

**Figure:**
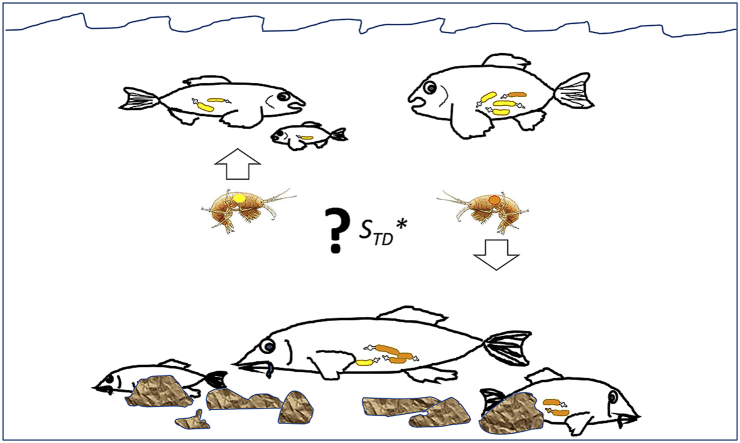

## 1 Introduction

Host specificity and infection patterns are fundamental concepts in the ecology and evolution of parasitism (Shaw and Dobson, 1995; Poulin, 2007; Poulin et al., 2011). Determinants of host specificity and patterns of host use include factors such as distribution, abundance and relative suitability of host species. For instance, parasite aggregation among hosts is expected to increase with inter-host differences in exposure or susceptibility to parasites (i.e. probability of encounter; Anderson and Gordon, 1982; Wilson et al., 2002; Poulin, 2007a), and with host specificity (i.e. host-parasite compatibility; Pérez-del-Olmo et al., 2011). Determinants of specificity and patterns of host use also include parasite species-specific features, such as life-cycle (direct or complex) and transmission mode (e.g. passive, active or trophic; Shaw and Dobson, 1995; Wilson et al., 2002; Poulin, 2007). Parasites with complex (i.e. heteroxenous) life-cycles and trophic transmission rely on the consumption of infected intermediate hosts by predators suitable as definitive hosts to complete their life-cycle. They are expected to accumulate in definitive hosts, especially those occupying higher trophic levels as these hosts are likely to consume large numbers of infected intermediate host prey (Pérez-del-Olmo et al., 2011; Lester and McVinish, 2016). Heteroxenous parasites are often expected to exhibit low specificity towards their definitive hosts compared to parasites with direct cycle and transmission by contact (Poulin, 2007). However, if trophic transmission is increased by parasite-induced behavioural alterations in the intermediate host, the range of definitive hosts actually used could be more restricted than expected as behavioural manipulation may target species-specific host foraging behaviours (Fredensborg, 2014). Yet, spatial distribution among hosts and variations in the level of host specialization are still poorly documented in heteroxenous parasites capable of host behavioural manipulation (Fredensborg, 2014).

In addition to complex determinants of specificity, poor taxonomic resolution in most parasite groups may hinder accurate estimations of host range (Poulin and Keeney, 2008; Locke et al., 2010, 2013; Poulin and Leung, 2010). This has been recently highlighted by the improved records of specificity and parasite community achieved using DNA barcoding (Smith et al., 2007; Steinauer et al., 2007; Locke et al., 2010; Lootvoet et al., 2013; Steinauer and Nickol, 2015). In addition, DNA barcoding can further our understanding of parasite historical biogeography, and provide new evidence for biological invasion and replacement (Hohenadler et al., 2017; David et al., 2018; Perrot-Minnot et al., 2018).

In this context, we used acanthocephalan parasites to address patterns of host use and specificity. Acanthocephalans have a two-host life-cycle involving arthropods as intermediate hosts and vertebrates as definitive hosts. Arthropods become infected when accidentally consuming parasite eggs. In the intermediate host, the parasite grows and then enters a dormant stage (cystacanth) infective to the definitive host. Upon consumption by the appropriate vertebrate definitive host, further growth, sexual maturation and reproduction take place; parasite eggs are then released with host faeces (Crompton, 1985). Trophic transmission to definitive hosts is generally enhanced by multiple behavioural alterations in infected intermediate hosts (Moore, 2002; Cézilly et al., 2013; Fayard et al. in prep.). Specificity towards definitive hosts is often expected to be low in acanthocephalans. First, their complex life-cycle has likely evolved by ‘upward incorporation’ of vertebrate predators that became the definitive hosts (Herlyn et al., 2003; Parker et al., 2003). Shorter coevolutionary history with definitive hosts than with intermediate hosts could translate into lower specificity, assuming a general trend towards increased specificity with increased coevolutionary time (Noble et al., 1989 in Poulin, 2007). Second, invertebrate species used as intermediate hosts are generally preyed upon by a wide range of predators. Numerous trophic links could offer multiple transmission opportunities, assuming that predators can be accommodated as paratenic or definitive hosts in the course of the parasites evolution. Third, selective pressure on vertebrate definitive hosts to avoid infected prey could be weak, because the benefits from preying upon more accessible ‘manipulated’ prey could balance the energetic costs of infection (Lafferty, 1999). Therefore, a large range of predators could be used as definitive hosts. However, the taxonomic revision of previously unresolved species complex occasionally revealed an underestimated level of specificity towards definitive hosts, as reported in two freshwater fish acanthocephalans (Steinauer et al., 2007; Martínez- Aquino et al., 2009; Steinauer and Nickol, 2015). For instance, the recognition of six species within the North American fish acanthocephalan *Leptorhynchoides thecatus* complex, based on DNA sequence data and morphological re-description, revealed specificity at the level of fish genus (Steinauer et al., 2007; Steinauer and Nickol, 2015).

The goal of the present study is precisely to determine patterns of host use and potential host specificity in two acanthocephalan parasites of freshwater fish, *Pomphorhynchus laevis* (Zoega in Müller, 1779) and *Pomphorhynchus tereticollis* (Rudolphi, 1809). Previous records of *Pomphorhynchus laevis sensu lato* Amin et al. (2003) suggest a wide geographic distribution throughout the Western Palaearctic area (Perrot-Minnot et al., 2018), a broad range of freshwater and brackish-water fish species as definitive hosts and a diversity of amphipods as intermediate hosts (Kennedy, 2006; Špakulová et al., 2011; Vardić Smrzlić et al., 2015). However, the recent taxonomic revision of *P. laevis s.l.* has revealed a confusion between *P. laevis* and *P. tereticollis,* and led to the erection of the latter as a true species (Špakulová et al., 2011). Since some species-specific features necessitate the careful examination of adult proboscis, which is more challenging on fixed than on fresh samples, *P. tereticollis* has previously been recorded as *P. laevis* in most parasitological surveys (Špakulová et al., 2011). Therefore, genetic studies are recommended to establish the geographic and ecological distribution of these two acanthocephalan species (Špakulová et al., 2011; Perrot-Minnot et al., 2018).

Here, we documented patterns of definitive host use by *P. laevis* and *P. tereticollis* within the fish communities of two different rivers/localities where they occur in sympatry. We relied on DNA-based species identification using the Internal Transcribed Spacer 1 gene (ITS1) to compare the distribution of *P. laevis* and *P. tereticollis* among fish hosts species, and addressed whether the composition of the local fish community affects the mean abundance of each *Pomphorhynchus* species within each host species (Arneberg et al., 1998; Canard et al., 2014). The estimated abundance of *P. laevis* and *P. tereticollis* within each fish species was used to calculate several indexes of specificity and to estimate niche overlap. We also contrasted the transmission efficiency of each *Pomphorhynchus* species within the local network of definitive hosts by quantifying their flow rate among fish species. We next addressed whether variable compatibility levels towards different fish species could account for the observed contrasted abundance in the two main hosts, barbel and chub, by quantifying parasite size and reproductive parameters. *Pomphorhynchus* spp. size is expected to increase markedly from larval stages (cystacanth, less than 1 mm diameter) to adults (from 5 to over 10 mm body length). Gonadal development and gametogenesis proceed in parallel to adult growth. Hence, both adult body size and reproductive potential are expected to increase with host quality, thereby indicating the level of compatibility towards final hosts. From this comparative study of host use, we discuss the potential causes of specificity of these two *Pomphorhynchus* species towards definitive hosts, at a local scale.

## 2 Material and methods

### 2.1 Localities, fish community composition and sampling

Two localities in eastern France were sampled in spring and summer, one on the river Ouche (47°17′54.56"N 5°2′21.97"E) in 2003 and 2005, and one on the river Vingeanne (47°20′51.66"N 5°27′8.76"E) in 2004 and 2005. We retrieved information on the composition of the local fish community in these two localities from the Agence Française pour la Biodiversité database (fish-based ecological assessment in the framework of EU Water Directive), based on the regular monitoring of fish species richness and abundance between 2001 and 2006. A total of thirty fish species were identified of which fourteen species were present in both localities (Fig. S1). The two localities differed in their fish community with a higher species richness, fish density and biomass in the Vingeanne locality (S = 24; 414 individuals per100 m^−2^; 15.5 kg per100 m^−2^) compared to the Ouche locality (S = 20; 93 individuals per 100 m^−2^; 1.84 kg per 100 m^−2,^ on average). Diversity of fish community was also higher in the Vingeanne locality (Shannon-Wiener index of diversity H’ = 3.44, estimated from 24 species and 2901 individual fish) than in the Ouche locality (H’ = 2.89, estimated from 20 species and 3511 individual fish).

Fish were captured by electric fishing or nets, killed immediately, identified, measured (fork length) and weighed. They were then dissected and their intestine removed to collect adult acanthocephalan individuals attached to the intestinal wall. Since the occurrence of paratenic hosts or dead-end hosts had been reported previously for *Pomphorhynchus laevis s.l.* (Médoc et al., 2011), we inspected fish body cavity and viscera for extra-intestinal infection with *Pomphorhynchus* cystacanths. Parasites from each fish were stored individually in absolute ethanol for molecular analyses. Only fish species for which at least five individuals were dissected were included in the analyses.

To compare the relative proportion of the two *Pomphorhynchus* species in each fish species to that in the intermediate host prey population, we collected a large sample of amphipods in each localities at the same time as fish sampling. In each river, 36 samples were taken every 3 m with a kick net (diameter 30 cm, mesh size, 500 μm), moving upstream. Infection status and gammarid species identification (*Gammarus pulex*/*G. fossarum* or *G. roeseli*) were performed on EtOH-preserved samples, using a stereoscopic microscope (Nikon SMZ-10A).

### 2.2 DNA-based species identification

Parasite species identification was performed based on a species-specific difference in the length of the first Internal Transcribed Sequence (ITS1) situated between the subunits 5.8 and 26S of nuclear ribosomal gene (Perrot-Minnot, 2004; Franceschi et al., 2008). DNA extraction used a three-step procedure including lysis in CTAB buffer, phenol-chloroform purification and isopropanol precipitation, following Perrot-Minnot (2004). PCR reactions were performed in a final volume of 10 μL containing 1 μL template DNA, 200 mM of each nucleotide, 5 pmol of each primer and 0.25 units of *Taq* DNA polymerase (HotMaster™ *Taq*, Eppendorf) in 1X HotMaster™ *Taq* buffer with 2.5 mM MgCl_2_ (Eppendorf). Primer sequences and amplification conditions are detailed in Franceschi et al. (2008). The size of generated amplification products was 320 bp for *P. laevis* and 350 bp for *P. tereticollis,* and was visualized through electrophoresis of 2 μL of PCR product in 1.5% agarose gel. We used both a DNA size standard (100 bp ladder, Fermentas) and a mix of *P. laevis* and *P. tereticollis* PCR products, as size-markers.

### 2.3 Infection patterns of *P. laevis* and *P. tereticollis*

We first established the proportion of *P. laevis* and *P. tereticollis* in each fish species and compared it to the one expected from their distribution in amphipod intermediate hosts. Because the number of individual parasites genetically identified per individual fish was low (median = 3, min-max = 1–43, 1^rst^ and 3rd quartiles = 1–5), we pooled all parasites collected from at least 5 individual hosts per fish species, and calculated the proportion of *P. laevis* and *P. tereticollis* harboured by each fish species. We also compared the relative proportion of *P. laevis* and *P. tereticollis* in each fish species according to fish ecology: benthic (bottom-feeders) or bentho-pelagic (i.e. drift-feeders; Oberdorff, 1995).

We then tested the hypothesis that differences in parasite abundance among fish species could be determined by host species abundance, by regressing log10-transformed mean parasite abundance per fish species on log10-transformed fish biomass (Arneberg et al., 1998; Buck and Lutterschmidt, 2017). We also illustrated the transmission efficiency of parasites within the local network of definitive hosts by estimating the flow rate of *P. laevis* and *P. tereticollis* among fish species. Flow rate was calculated as the product of the mean *P. laevis* or *P. tereticollis* abundance per fish species and the density of each fish host species in the community.

Host diversity and specificity were quantified for each *Pomphorhynchus* species using three indices: Shannon - Wiener's diversity index (H′) as a measure of structural host diversity, phylo-structural index of specificity *S*_TD_* (Poulin and Mouillot, 2005; Poulin et al., 2011), and Paired Difference Index (PDI) as a quantitative measure of specialization (Poisot et al., 2012). The Shannon-Wiener index was calculated from relative host use by *P. laevis* and *P. tereticollis*, i.e. their relative abundance within each fish species. The phylo-structural index of specificity, combining the taxonomic hierarchy of hosts to observed prevalence, was calculated after Poulin and Mouillot (2005). This index varies with the number of host species and the prevalence in each species, according to the taxonomic distance between species: increasing the number of species with taxonomic redundancy decreases *S*_TD_* while adding species with a distant taxonomic position increases *S*_TD_*, especially at high prevalence. Therefore, *S*_TD_* index is inversely proportional to specificity. To calculate *S*_TD_*, we used six levels of taxonomic hierarchy (Genus -all distinct-, Subfamily, Family, Order, supra-Order, and infra-Class Teleostei). Within the Order Cypriniform, we obtained information about host's taxonomic rank from Gaubert et al. (2009). The Paired Difference Index contrasts a species' strongest link on a resource with those over all remaining resources (Poisot et al., 2012; R package ‘bipartite’, Dormann et al., 2017). Here we used fish species as resource, and the relative mean abundance of intestinal *P. laevis* or *P. tereticollis* as a measure of link strength between parasite and fish host. The Paired Difference Index ranges from 0 (generalist) to 1 (perfect specialist; Poisot et al., 2012). Finally, the degree of niche overlap between the two *Pomphorhynchus* species was estimated by calculating Renkonen's similarity index. This index was chosen based on the recommendations of Wolda (1981) for samples with contrasted abundance. It was calculated using the relative flow rate of each *Pomphorhynchus* species among fish host species as a descriptor of parasite niche, after log-transformation.

To assess relative compatibility towards fish hosts, we focused on the two main hosts of *P. laevis* and *P. tereticollis*, based on their use (mean abundance) and on their local abundance within the fish community: the barbel *Barbus barbus* and the European chub *Squalius cephalus.* We estimated worm development by measuring parasite size (body length) on thawed worms in water, from photographs taken under a stereomicroscope (SMZ 1500, Nikon) and using the image analysis software LUCIA G 3.81. We also recorded the position of each individual worm along the intestinal tract. We then measured several reproductive parameters to assess male and female reproductive success according to parasite and fish species. We measured testes volume in adult males (Fig. S3c), and, the number and volume of ovarian balls (free ovaries) and the number of eggs in females. Ovarian balls are produced by ovarian fragmentation in the definitive host, and increase in size and cell number during their development, before the progressive release of mature oocytes upon insemination (Crompton, 1985). Therefore the number of ovarian balls and the number of eggs might well reflect host suitability to sustain ovarian development (Crompton, 1985). Testes volume and the number and volume of ovarian balls were measured from photographs. The ovoid volume (testes and ovarian balls) was estimated from length and width measurements using the formula of ellipsoid volume (V = π*L*D^2^/6; L: length, D: diameter). We collected eggs from female body cavity and stored them in 200 μl of 10% formaldehyde at 8 °C. The number of eggs in the suspension was estimated using an automatic particle counting machine (Coulter^®^ Multisizer™). Egg size distribution was automatically partitioned into 70 size categories ranging from 11.55 μm to 33.59 μm, revealing three clusters of size categories gathering more than 65% of total egg number (peak 1, 2, 3 in Fig. S3a).

### 2.4 Data analysis

All statistical analysis were performed with R software (v 3.5.1.).

We compared the relative proportion of *P. laevis* and *P. tereticollis* in each fish species to that expected from their relative proportion in amphipod intermediate hosts using Fisher's exact test for count data. Bonferroni correction for multiple tests was applied to critical threshold value (number of fish species in each locality, Ouche, N = 12, Vingeanne, N = 6, including fish with extra-intestinal parasites). In addition, we tested the effect of fish ecological type – benthic *versus* bentho-pelagic – and population, on the relative proportion of *P. laevis* and *P. tereticollis,* using a GLM with binomial distribution, and ecological type nested within population.

We performed a linear regression on log (10)-transformed variables to analyse the relationship between mean abundance of each *Pomphorhynchus* species and fish biomass, across fish species. We used linear mixed model regression (R-package *‘lme4’*; Bates et al., 2015) to analyse variations in parasite body size according to fish host species, parasite species and their interaction, population, and parasite load (the number of parasites per individual fish), with individual fish identity included as a random factor. We incorporated *Pomphorhynchus* load (i.e. number of individual parasites per host) as a predictor of parasite size to account for potential density-dependent effects (Crompton, 1985). We first performed a PCA on female reproductive parameters to avoid multicollinearity and to identify potential relationships among the six variables – number and volume of ovarian balls, number of eggs, proportion of eggs in the three size categories - (R-package *‘FactoMinR’*, v. 1.41; Lê et al., 2008). We used linear mixed models (lmer) to analyse variation in testes volume (cubic root), and coordinates on the first two principal components (female reproductive parameters), according to parasite size (log-transformed), fish host species, parasite species, the interaction of both, and population, with fish identity included as a random factor. We then performed model comparison to estimate the contribution of each predictor variable to variation in size and reproductive parameter. The approach is based on deviance comparison between models fitted to the same data - the full model and the model without one predictor variable- (maximum likelihood ratio test, R-package ‘*lmtest’*, Zeileis and Hothorn, 2002). We used the associated Chi-square value and probability to assess the significance of predictor variable.

## 3 Results

*Pomphorhynchus* samples were collected from 14 species of fish, mainly cyprinids, among which 4 species were collected in both localities (Fig. S1). A total of 881 fish were dissected; 752 from 11 fish species sampled in the Ouche River and 122 from 7 fish species sampled in the Vingeanne River. Before running the analysis, we verified that the sampling of each fish species was representative of the local fish community, irrespective of whether the fish species hosted *Pomphorhynchus* parasite or not (Spearman rank correlation between the relative abundance of each fish species within our sample, and the relative abundance of each species within the local community: Ouche: S = 272, *P* < 0.0001, N = 20, Rho = 0.80; Vingeanne: S = 862.9, *P* < 0.0001, N = 26, Rho = 0.70; Fig. S1). Fish species reported at relative densities lower than 1.5% were not sampled, except for stickleback, rudd and perch in the Ouche River, and catfish in the Vingeanne River. All fish species reported at a relative density higher than 1.5% were sampled, except for spirlin and minnow (Table S1; Fig. S1). Among the sampled fish, 550 (both infected and uninfected) were kept for parasite analysis (Table S1).

### 3.1 Host range and specificity of *P. laevis* and *P. tereticollis*

All parasites sampled in the Ouche locality and almost all parasites from the Vingeanne locality were successfully assigned to one of the two *Pomphorhynchus* species (with only one individual fish out of 10 having less than 80% of its parasites assigned to either species in the Vingeanne locality). Identification effort per individual fish was representative of parasite intensity, as confirmed by a significant correlation between the number of parasites identified and *Pomphorhynchus* intensity (spearman rank correlation test, S = 67546, *P* < 0.0001, Rho = 0.89). We also genotyped 95 extra-intestinal *Pomphorhynchus* from 33 individual fish from 4 species in the River Ouche, and from 3 catfish individuals in the River Vingeanne, out of the 475 extra-intestinal parasites recorded (20%).

**Fig. 1:**
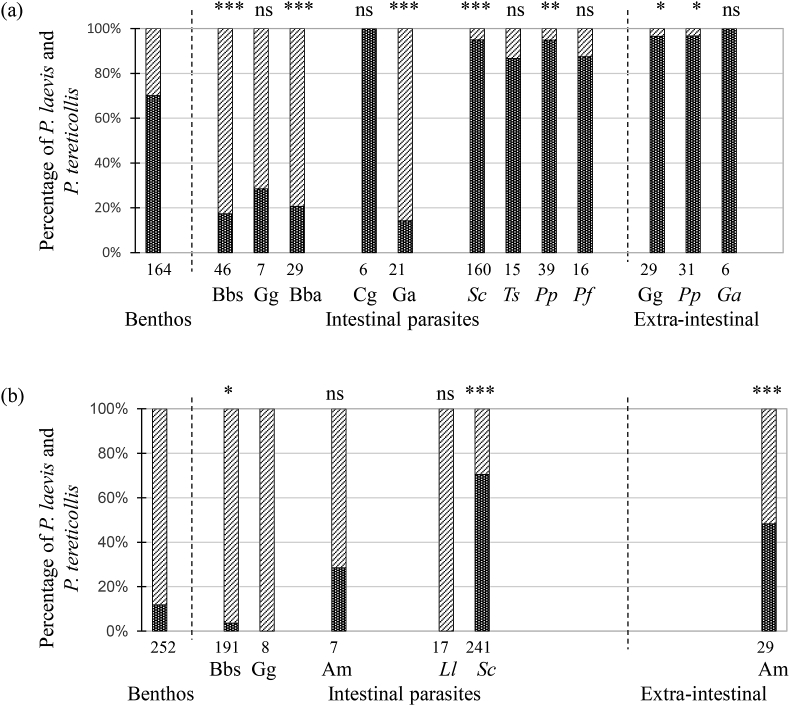
Percentage of *P. laevis* (plain bars) and *P. tereticollis* (striped bars) among *Pomphorhynchus* parasites collected from amphipod intermediate hosts in the benthos, and from the fish definitive hosts, either in the intestine or collected as extra-intestinal cystacanths, in (a) the Ouche and (b) Vingeanne localities. The proportion of *P. laevis* and *P. tereticollis* in each fish species was compared to their proportion in intermediate gammarid hosts using Fisher exact test (*P*-values given after correction for multiple test: ***: P < 0.001; **: P < 0.01; *: P < 0.05; ns: not significant). Letters above bars refer to post-hoc comparison between fish species (Tukey HSD). Numbers below bars are sample sizes (number of parasites). Bentho-pelagic fish species are abbreviated in italics, benthic species in standard font. Species abbreviations: *Barbus barbus,* Bbs; *Gobio gobio,* Gg; *Squalius cephalus,* Sc; *Telestes souffia,* Ts; *Chondrostoma nasus,* Cn; *Rutilus rutilus,* Rr; *Scardinius erythrophthalmus,* Se; *Leuciscus leuciscus,* Ll; *Phoxinus phoxinus,* Pp; *Barbatula barbatula,* Bba; *Ameiurus melas,* Am; *Perca fluviatilis,* Pf; *Cottus gobio,* Cg; *Gasterosteus aculeatus,* Ga.

Across all fish species, *P. laevis* represented 71.3% and 37.8% of all intestinal acanthocephalan parasites genotyped from the Ouche (N = 342) and Vingeanne River (N = 473), respectively. A higher proportion of *P. laevis* than *P. tereticollis* was found as extra-intestinal parasites, reaching 97% and 48% of extra-intestinal individuals in fish from Ouche and Vingeanne localities, respectively. Extra-intestinal parasites from gudgeon, minnow and stickleback were nearly all *P. laevis,* while catfish harboured both *P. laevis* and *P. tereticollis* as extra-intestinal cystacanths (Fig. 1).

**Fig. 2:**
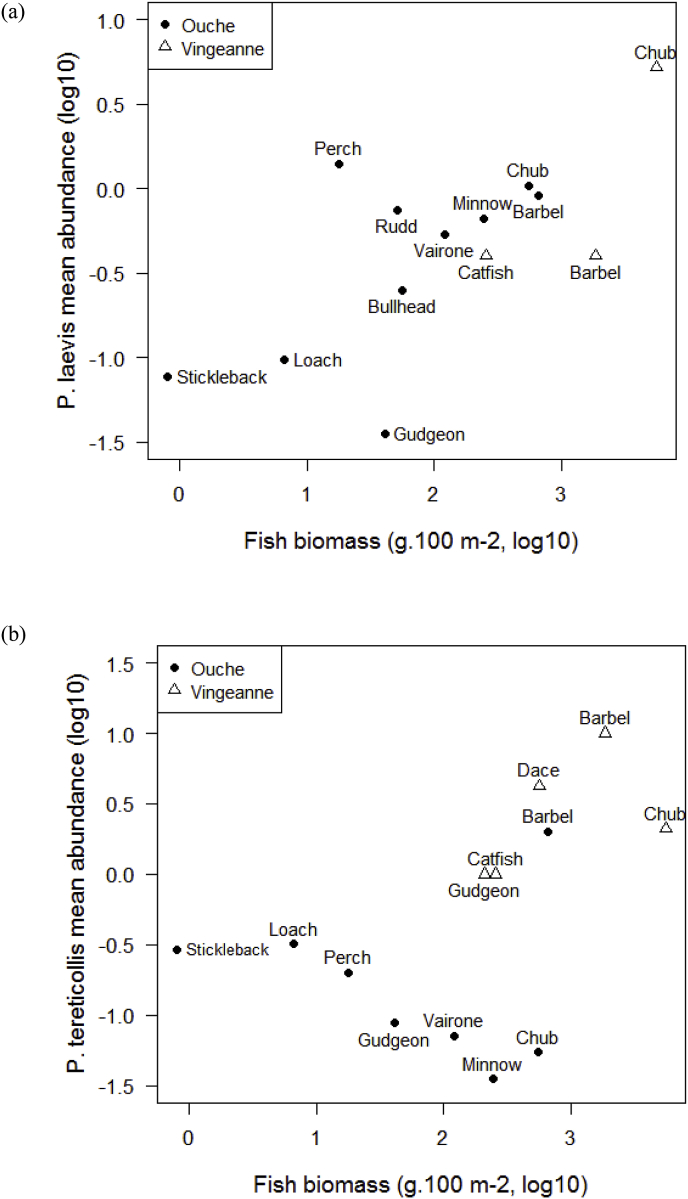
Relationship between fish biomass (g.100 m^-2^) and *P. laevis* (a) or *P. tereticollis* (b) mean abundance per fish species, across fish species and localities (Ouche and Vingeanne localities). Both variables were log10-transformed.

To test whether the relative proportion of each parasite species in fish hosts differs from the proportion found in intermediate hosts, we first estimated the proportion of *P. laevis* and *P. tereticollis* in pooled samples of gammarids collected in October 2004, February 2005 and May 2005. Overall, 195 out of 7290 gammarids from the river Vingeanne (2.67%) and 145 out of 5737 from the river Ouche (2.5%) were infected with *Pomphorhynchus* cystacanths. The relative number of gammarids infected with *P. tereticollis* and *P. laevis* was different between the two localities (Fisher exact test, Chi^2^ = 105.85, df = 1, *P* < 0.0001). Prevalence of *P. laevis* in gammarid hosts was higher than that of *P. tereticollis* in the Ouche locality (1.67% and 0.85%, respectively), while the reverse was found in the Vingeanne locality (0.32%, and 2.34%, respectively; Fig. 1). In most fish species from which at least 20 *Pomphorhynchus* were genotyped, one of the two species was significantly more abundant than expected from its relative abundance in intermediate hosts (Fisher exact test, probabilities corrected for multiple testing: *P* < 0.05 to 0.001; Fig. 1). In addition, the relative proportion of *P. laevis* and *P. tereticollis* differed significantly according to fish ecology (nested GLM with binomial distribution: Population effect, df = 1, Chi^2^ = 85, *P < *0.0001, ecological type nested within population, df = 2, Chi^2^ = 398.5, *P* < 0.0001). More specifically, *P. laevis* was relatively more abundant in bentho-pelagic fish, in particular chub in both localities, and vairone and minnow in the river Ouche. .Conversely, *P. tereticollis* was relatively more abundant in benthic fish (bottom-feeders) in both localities, in particular barbel in both localities (and loach in the river Ouche; Fig. 1). The mean abundance of *P. laevis* in each host species increased significantly with local fish biomass (Rsq.adj. = 0.41, F_1,11_ = 9.1, *P = *0.012; Fig. 2). This trend was not significant for *P. tereticollis* (Rsq.adj. = 0.07, F_1,9_ = 1.36, *P = *0.21), with bentho-pelagic fish from the Ouche River harbouring low abundance of *P. tereticollis* relative to their local biomass (particularly chub and minnow; Fig. 2).

Host range structural diversity (Shannon-Wiener index H’) was higher, and taxonomic diversity (phylo-structural index *S*_TD_*) and specialization (link strength on resource: PDI) lower, for *P. laevis* compared to *P. tereticollis* in the Ouche locality, while the reverse was found in the Vingeanne locality (Table 1). The higher strength of link on resource (PDI) in Vingeanne locality compared to Ouche locality was associated with a lower structural diversity (Shannon index), independently of parasite species. Interestingly, the prevalence in intermediate hosts increased with increasing taxonomic specificity towards fish hosts (lower phylo-structural index *S*_TD_*), irrespective of locality and *Pomphorhynchus* species (Table 1; Fig. 4). Weak negative or positive associations between prevalence in intermediate host and resource specialization or structural diversity, respectively, were also observed (Table 1). The level of niche overlap between *P. laevis* and *P. tereticollis* was low to intermediate in both localities (Table 1), and lower in the Vingeanne than Ouche locality.

**Table 1:** Indexes of structural diversity (Shannon-Wiener), specificity (phylo-structural index), and link intensity on resource (PDI), of two acanthocephalan parasites of fish, *Pomphorhynchus laevis* and *P. tereticollis*, and level of niche overlap between the two parasites. Parameters of host use (species range and mean abundance per host species) were estimated from a large sample of fish representative of local fish community, in two rivers where the two parasite species occur in sympatry (Ouche and Vingeanne rivers).

	Shannon index of diversity		Phylo-structural index		Paired Difference Index		Niche overlap between *P. laevis* and *P. tereticollis*
	*P. laevis*	*P. tereticollis*	*P. laevis*	*P. tereticollis*	*P. laevis*	*P. tereticollis*	Renkonen’ index (Log flow rate)
Ouche	**2.85**	1.77	0.69:	**0.74**	0.71	**0.94**	**0.33**
Vingeanne	0.44	**1.6**	**0.82**	0.61	**0.96**	0.92	0.21

Significant values are indicated in bold.

**Fig. 3:**
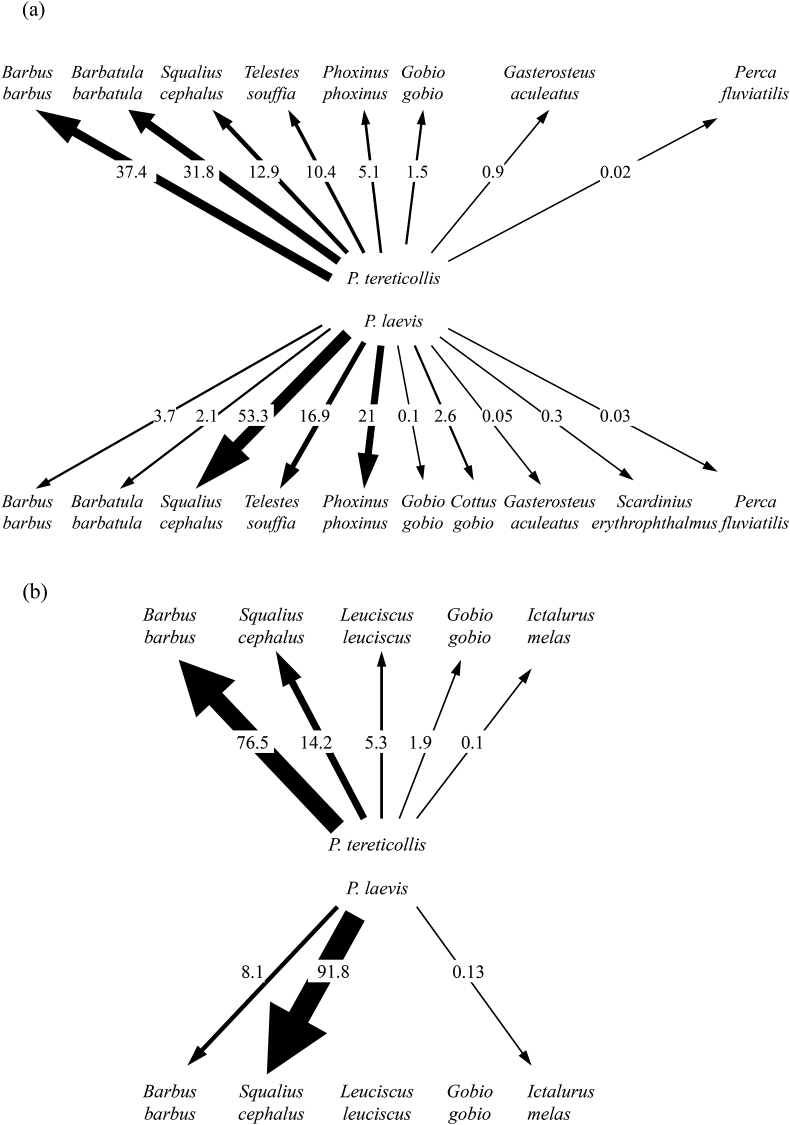
Relative flow rates of *P. tereticollis* and *P. laevis* in their hosts in the Ouche (a) and Vingeanne (b) localities, taking into account the abundance of adult intestinal parasites and the relative abundance of each fish host species in the local community. The thickness of arrows and percentages indicate the estimated relative value of each fish species as a resource for each *Pomphorhynchus* species.

Combining parasite abundance to the local density of each fish species revealed that 80% (Ouche locality) to 90% (Vingeanne locality) of intestinal *P. laevis* and *P. tereticollis* were cycling through only one to three host species (Fig. 3a and b). As illustrated by their respective flow rate, the two *Pomphorhynchus* species exhibited a contrasting use of two of the most abundant fish species, with *P. laevis* mainly using European chub and *P. tereticollis* mainly using common barbel. This pattern was particularly clear in the Vingeanne locality, where fewer fish species were used overall (Fig. 3b).

**Fig. 4:**
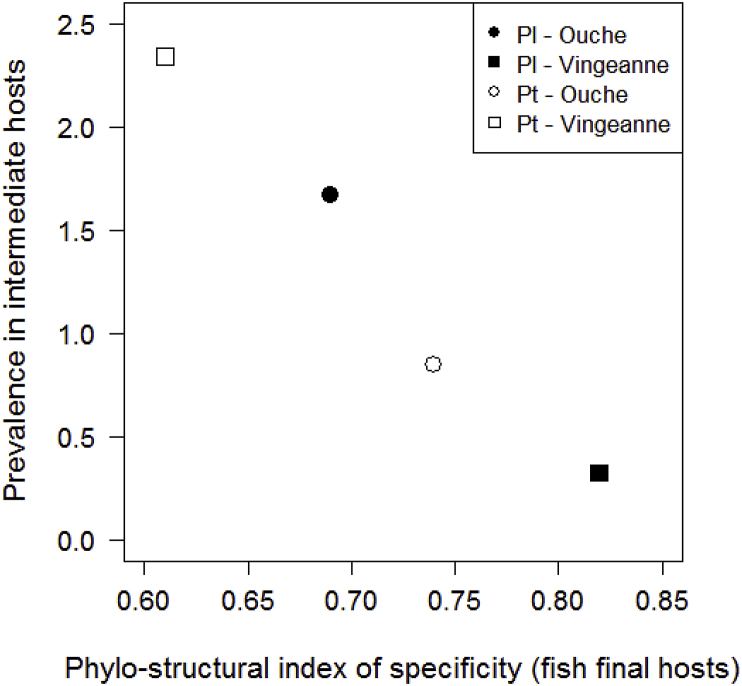
Relationship between the phylo-structural index of specificity towards definitive hosts and observed prevalence in gammarid intermediate hosts (%) in *P. laevis* and *P. tereticollis*. The phylo-structural index of specificity increases with the taxonomic diversity of fish species used.

### 3.2 Parasite size and reproduction in barbel and chub

**Fig. 5:**
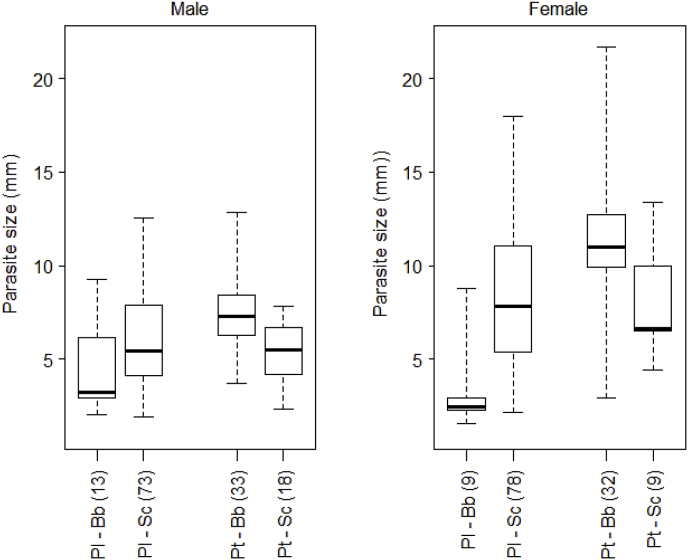
Intestinal *Pomphorhynchus* size (body length in mm) according to parasite species (Pl, *P. laevis* and Pt, *P. tereticollis*), fish host species (Bb, *Barbus barbus*; Sc, *Squalius cephalus*) and parasite sex (sample size is given into brackets. total sample size N = 265).

Variation in parasite body size was analysed on 265 intestinal worms. Overall, parasite size was independent of *Pomphorhynchus* load, and of the type of infection – monospecific or mixed infection - in individual fish. Females worms were larger than males (Table 2a; Fig. 5). *Pomphorhynchus laevis* was significantly larger in chub than in barbel, while *P. tereticollis* tended to be larger in barbel than in chub, although not significantly (Table 2a; Fig. 5). Host effect was generally stronger on female than male size (Fig. 5). Both species were found occupying a restricted region of the intestinal tract (within the first part of the second-third section) with no evidence for microhabitat segregation between them (Fig. S2). Their distribution along the intestinal tract did not change under heterospecific infection as compared to monospecific infection.

**Table 2:** Analysis of parasite size and reproductive parameters according to parasite species, fish species, their interaction, population, and, for parasite size, *Pomphorhynchus* load (the number of parasites per individual fish) and sex, and for reproductive parameters parasite size (a) Parasite size, and male testes volume; (b) female reproductive parameters, represented by the two first PCA axis (main contributing variable provided; see Fig. S3b). Infection type refers to monospecific or heterospecific infection with respect to *Pomphorhynchus* species.

(a)		
Dependant variable Predictor variable	Parasite size Chi^2^ (df; *P* value)	Testes Chi^2^ (df; *P* value)
Parasite load	0.8 (1; *P* = 0.37)	
Parasite sex	**24.1 (**1; ***P* < 0.0001)**	**/**
Parasite size	/	**137.8 (1; *P* < 0.0001)**
Fish sp.	***P.laevis*: 8.67 (1; *P* = 0.003)***P. tereticollis:* 2.69 (1; *P* = 0.10)	5.3 (2; *P* = 0.07)
Parasite sp.	***in barbel:* 44.5 (1; *P* < 0.0001)***in chub:* 0.09 (1; *P* = 0.76)	1.5 (2; *P* = 0.47)
Fish * Parasite sp.	**34.8 (1; *P* < 0.0001)**	0.02 (1; *P* = 0.87)
Infection type ^$^	2.01 (1; 0.15)	/
Population	1.6 (1; *P* = 0.20)	0.4 (1; *P* = 0.63)

(b)		

Dependant variable Predictor variable	PC 1 ∼ nb eggs, prop.egg3 Chi^2^ (df; *P* value)	PC2∼ nb ovarian balls Chi^2^ (df; *P* value)

Parasite size	**43.4 (1; *P* < 0.0001)**	0.72 (1; *P = *0.39)
*P. laevis* in chub *vs* *P. tereticollis* in barbel	**21.3 (1; *P* < 0.0001)**	**4.33 (1; *P* = 0.04)**
Population	0.1 (1; *P* = 0.31)	3.53 (1; *P = *0.06)

Significant values are indicated in bold.

**Fig. 6:**
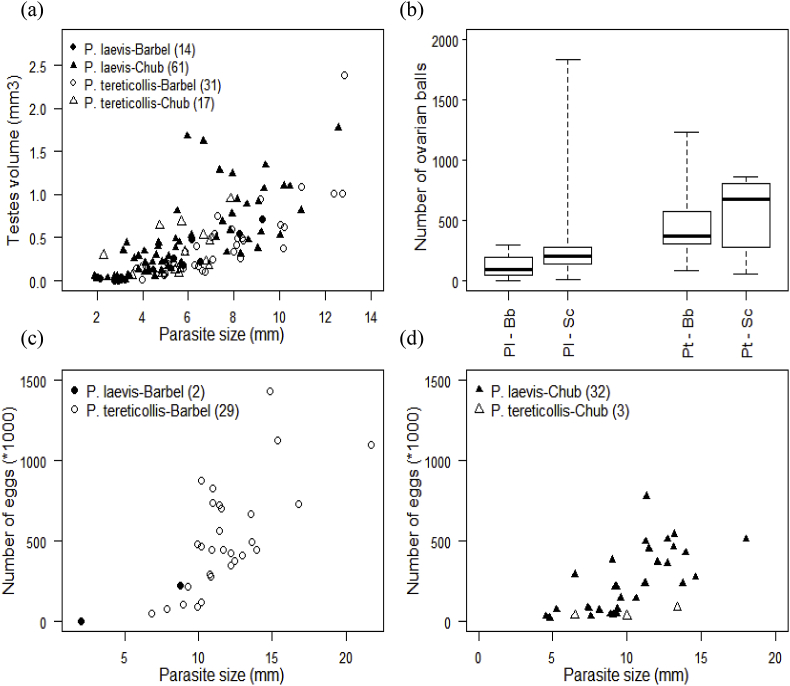
Reproductive parameters of *P. laevis* and *P. tereticollis*: (a) testes volume as a function of adult worm size, according to parasite species and fish host; (b) number of ovarian balls according to parasite species and fish host; (c, d) number of eggs as a function of female size according to parasite species, in barbel (c) and chub (d).

Testes volume was positively correlated with male size, and did not differ between parasite species nor fish host species (Table 2a; Fig. 6a). The first two principal components of PCA on female reproductive parameters retained 57.7% of the variance (Fig. S3b). Egg number and the proportion of eggs in the largest size category grouped together on the first axis, at the opposite of the volume of ovarian balls (Fig. S3b). The number of ovarian balls and the proportion of eggs in the smallest size category grouped together on the second axis (Fig. S3b). Female reproductive parameters could only be compared between *P. laevis* from chub and *P. tereticollis* from barbel, due to too low number of females in the other two host-parasite combinations. Variation in the two principal components was explained by parasite - host pair: *P. tereticollis* in barbel produced more eggs and ovarian balls than *P. laevis* in chub, independently of body size (Table 2b; Fig. 6c and d). The first principal component was also positively correlated with female size.

## 4 Discussion

Almost all fish species sampled in the two local fish communities harboured *Pomphorhynchus* parasites, with variable levels of infection. Despite this broad array of potential hosts, the actual host range of *P. laevis* and *P. tereticollis* showed limited overlap, as revealed by their relative flow rate within fish communities. In addition, the ratio of *P. laevis* to *P. tereticollis* abundance in most fish host species was either higher or lower than that expected from their local abundance in intermediate host prey. This pattern is indicative of transmission bias resulting from differential encounter rate with infected prey and compatibility towards fish host (Combes, 2001; Kuris et al., 2007). Interestingly, the host range of the two *Pomphorhynchus* species partially matched distinct fish ecology, with *P. laevis* infecting mainly bentho-pelagic fish and *P. tereticollis* mainly benthic fish species. To our knowledge, this is the first case of distinct host ranges partly matching the ecology of definitive hosts in closely related, sympatric acanthocephalans. Such trophic-transmission bias and associated niche distinctiveness between the two sympatric *Pomphorhynchus* species could only be uncovered based on recent taxonomic and molecular studies (Špakulová et al., 2011; Perrot-Minnot et al., 2018). Additionally, some species of fish harbored extra-intestinal parasites, and may act as dead-end or paratenic hosts. Small fish such as minnow, sticklebacks and gudgeon may act as paratenic hosts as shown experimentally by Médoc et al. (2011). Conversely, catfish are most likely dead-end hosts for both *P. laevis* and *P. tereticollis*, although a few worms established in the intestine. At low abundance (1.6% of total fish biomass), this invasive fish species still represents a negligible sink effect for parasites (here, less than 0.15% of adult worms).

Ecological indexes calculated for *P. laevis* and *P. tereticollis* in the two localities allowed further comparisons of their patterns of host use. Estimates of phylo-structural index of specificity reported here (from 0.61 to 0.82) were rather high, and therefore specificity rather low, compared to other fish acanthocephalans (from 0.37 to 0.58, in Muñoz and Cortés, 2009; and 0.47 and 0.55 in Rosas-Valdez and Pérez-Ponce de León, 2011), and to helminth parasites of bird (Poulin and Mouillot, 2004). This result could be due to the extensive sampling of local fish community performed in the present study, or to the use by the two *Pomphorhynchus* species of the largest freshwater fish family, Cyprinidae. Interestingly, specialization on resource was higher, and the structural diversity of host range lower, in the locality with the highest fish density and biomass (Vingeanne river). It is therefore possible that higher ecological opportunities for parasite transmission allow increased specialization of both *Pomphorhynchus* species, although a larger number of population replicates would be necessary to confirm this pattern.

Prevalence in intermediate hosts was negatively correlated to phylo-structural taxonomic specificity, irrespective of locality and parasite species. The interpretation of this pattern critically relies on identifying the causes underlying variation in prevalence. The high prevalence of cystacanths in gammarid hosts could reflect either a high overall density of parasites (high population growth rate: R0 hypothesis), or a low predation rate on cystacanth-infected prey (accumulation hypothesis). Under the first hypothesis, the lower prevalence in intermediate hosts with decreased taxonomic specificity (increased *S*_TD_*) would suggest the existence of a trade-off between the taxonomic diversity of host species used by one *Pomphorhynchus* species, and its overall efficiency in propagule production from final hosts. This would fit the widely recognized pattern in ecology known as the ‘jacks of all trades are often masters of none’, whereby the cost of being a generalist in host use is imposed on host colonization/infection success by parasites (Poulin and Mouillot, 2004), or, as suggested here, on propagule output (efficiency in host exploitation). Alternatively, following the second hypothesis of accumulation of cystacanth-infected gammarids under low predation rate, the lower prevalence in intermediate hosts with decreased taxonomic specificity would suggest an increased efficiency in trophic transmission when encountering a diverse range of fish host. A more thorough assessment of prevalence should therefore systematically include non-infective stages of *Pomphorhynchus* to get an overall estimate of parasite density. This is necessary to unravel the process underlying the relationship between host range and parasite specialization in definitive hosts, and parasite prevalence in intermediate hosts.

We further addressed one of the two main and non-exclusive hypotheses for such specialization, based on the encounter/compatibility paradigm (Combes, 2001; Kuris et al., 2007). We investigated whether variable levels of compatibility towards fish species was involved in niche segregation between *P. laevis* and *P. tereticollis*, by quantifying parasite development and reproduction. We focused on the two main hosts, barbel and chub, through which 50%–90% of adult parasites flow in the present localities. Body size of *P. laevis* was lower in barbel compared to chub independently of sex and population, while the same trend was found non-significant in *P. tereticollis.* We didn't find evidence for interspecific competition between the two parasite species, as infection type – monospecific *versus* heterospecific-had no effect on parasite size nor on parasite relative position along the intestinal tract. There was no evidence for a decreased male reproductive success in these suboptimal hosts neither. Male gonad size in *P. laevis* and *P. tereticollis* collected from barbel and chub reached comparable levels, after controlling for worm size. However, the development of testes is assumed to be largely completed at the cystacanth stage, within intermediate hosts (Crompton, 1985). Therefore, it is likely that fish host species had only little effect on testes size. We were not able to quantify female reproductive success in suboptimal hosts, due to a too low number of *P. laevis* females in barbel and of *P. tereticollis* females in chub, but *P. tereticollis* had a higher fecundity. Overall, these results suggest that compatibility is partial in particular for *P. laevis*, although a larger sample size would be necessary to better assess compatibility, especially on female reproductive success. It is still not clear whether the smaller adult size of *P. laevis* in barbel and of *P. tereticollis* in chub is indicative of occasional establishment failure as well (lower establishment success), or if all worms reaching final hosts established but failed to grow at the same rate. In other word, is the compatibility filter strong enough to explain the pattern of specialization, or is differential encounter rate also involved? One possible clue for decreased encounter rate also contributing to transmission bias is the apparent niche segregation of both *Pomphorhynchus* species between benthic and bentho-pelagic fish. Under this scenario, infected prey may segregate in different microhabitat according to parasite species. This is turn may modulate encounter rates with benthic and bentho-pelagic fish, and thereby partly account for distinct host range. Parasite-induced alterations of intermediate host behaviour have been widely documented in acanthocephalan parasites (Moore, 2002; Fayard et al. in prep.), in particular in *P. laevis* and *P. tereticollis* (Perrot-Minnot et al., 2007, 2014; Cézilly et al., 2013; Labaude et al., 2017). Under the hypothesis of encounter filter, increased vulnerability of infected gammarids to either benthic or bentho-pelagic fish according to *Pomphorhynchus* species would be explained by subtle differences in phenotypic alterations between the two parasite species. Differences in manipulation patterns may lie in the magnitude of behavioural changes, such as weaker alterations of phototaxis and geotaxis in *P. tereticollis* compared to *P. laevis* (Perrot-Minnot, 2004; unpublished data) or in distinct behavioural responses to fish olfactory cues. Whether such fine-tuned manipulation – if confirmed – has evolved to match the foraging behavior and micro-habitat of the most compatible fish hosts, or whether the use of definitive hosts with distinct ecology is the consequence of different patterns of parasite manipulation of intermediate hosts by the two *Pomphorhynchus* species, will not be easily established however. It is akin to the ‘chicken-and-egg’ problem raised by Cézilly et al. (2010), applied here at the level of closely related and sympatric acanthocephalan species.

## 5 Conclusion

Overall, our study challenges the common assumption of low levels of specificity towards definitive hosts in parasites with trophic transmission. In addition, host ranges of *P. laevis* and *P. tereticollis* exhibited low overlap in sympatry, which suggests distinct trophic transmission routes from shared intermediate host species, and/or variable compatibility levels according to fish hosts. This result raises the intriguing question of the determinants of definitive host segregation between sympatric *P. laevis* and *P. tereticollis*. It calls for a thorough assessment of the behavioural alterations induced by these two closely related species in their shared intermediate host, and of their relative establishment and reproductive success in final hosts. It also illustrates the necessity to improve taxonomic resolution in estimates of host specificity.

## Data accessibility

Data are available from the Mendeley repository: https://doi.org/10.17632/m3xmvybnzm.2.
