# Supplementary material for: Differential patterns of definitive host use by two fish acanthocephalans occurring in sympatry: Pomphorhynchus laevis and Pomphorhynchus tereticollis
Source: Int J Parasitol Parasites Wildl. 2019 Feb 1;8:135–44. doi: 10.1016/j.ijppaw.2019.01.007 (PMC6370571; doi:10.1016/j.ijppaw.2019.01.007)
Supplement: Multimedia component 1 [file mmc1.docx]

Differential patterns of definitive host use by two fish acanthocephalans occurring in sympatry: *Pomphorhynchus laevis* and *Pomphorhynchus tereticollis*

Marie-Jeanne Perrot-Minnot, Emilie Guyonnet, Loïc Bollache, Clément Lagrue

SUPPLEMENTARY INFORMATION

Fig. S1. Composition of the local fish community from the two localities sampled

Table S1: Number of individual fish per host species included in the dataset.

Fig. S2. Distribution of adults of *Pomphorhynchus laevis* and *Pomphorhynchus tereticollis* along the intestinal tract of barbels and chubs.

Fig. S3: Parasite reproductive parameters.


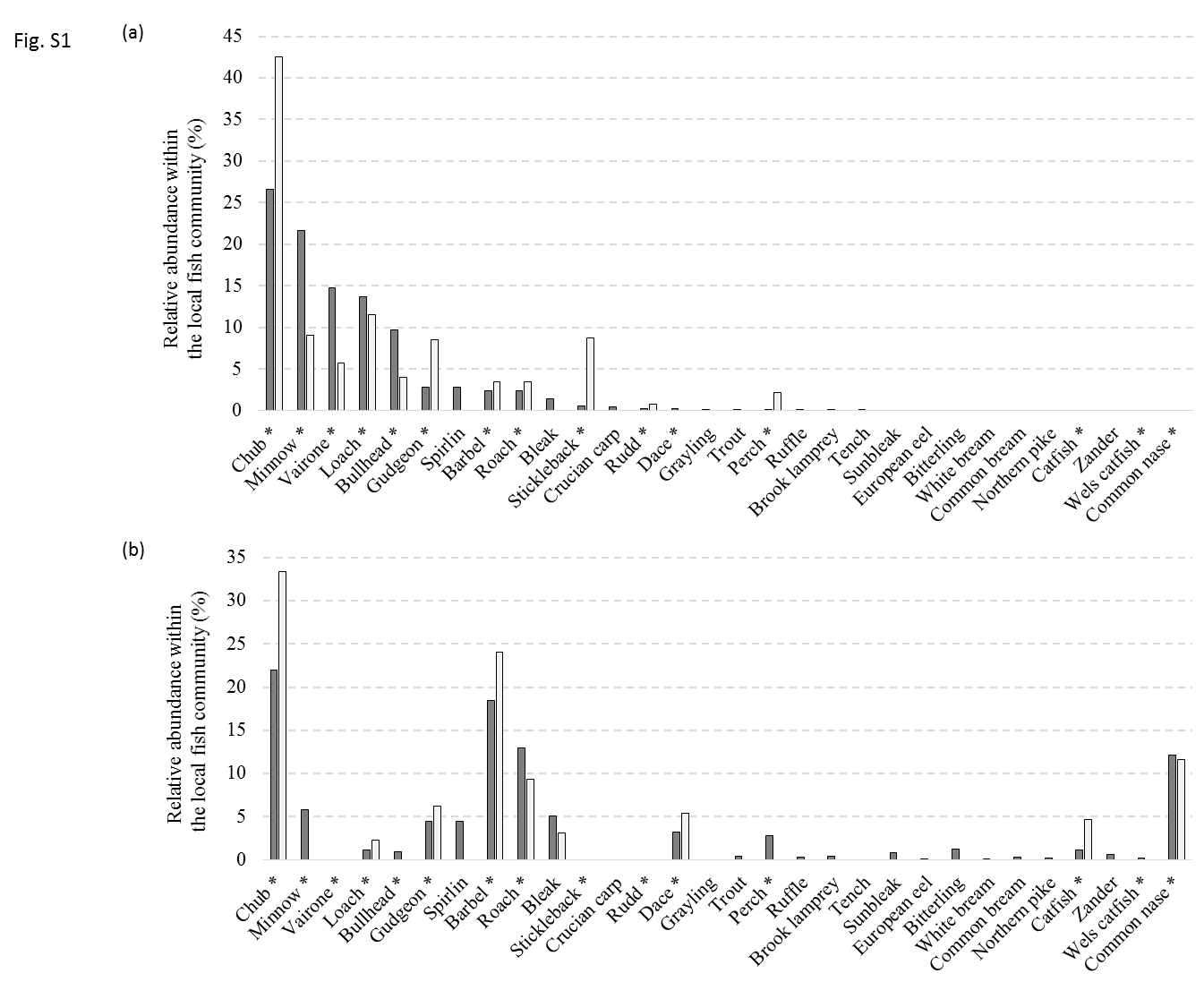


Fig. S1. Composition of the local fish community from the two localities sampled, in river Ouche (a) and river Vingeanne (b). The relative abundance of each fish species (dark grey) is estimated based on an extensive and long-term monitoring of the Talmay locality on River Vingeanne (N = 2901), and of two localities on River Ouche (Barbirey and Fauverney, upstream and downstream of the locality sampled, respectively) (N=3511) (source: Agence Française pour la Biodiversité). The relative abundance within the fish sample analyzed in this study is shown in light grey.

Table S1: Number of individual fish per host species included in the dataset, from two localities on rivers Ouche and Vingeanne (Eastern France), according to their infection status.

| Status Locality | Uninfected | Infected by  *P. laevis* | Infected by *P. tereticollis* | Mixed infection |
| --- | --- | --- | --- | --- |
| *Ouche* |  |  |  |  |
| Barbel | 0 | 2 | 6 | 5 |
| Vairone | 24 | 3 | 0 | 1 |
| Bullhead | 20 | 4 | 0 | 0 |
| Chub | 116 | 25 | 0 | 6 |
| Stickleback | 53 | 2 | 8 | 2 |
| Gudgeon | 54 | 0 | 2 | 1 |
| Loach | 54 | 4 | 11 | 3 |
| Perch | 6 | 3 | 1 | 0 |
| Rudd | 2 | 2 | 0 | 0 |
| Minnow | 50 | 5 | 0 | 2 |
| *Vingeanne* |  |  |  |  |
| Barbel | 2 | 0 | 14 | 4 |
| Chub | 3 | 15 | 2 | 15 |
| Gudgeon | 5 | 0 | 3 | 0 |
| Catfish | 4 | 0 | 0 | 2 |
| Dace | 1 | 0 | 3 | 0 |


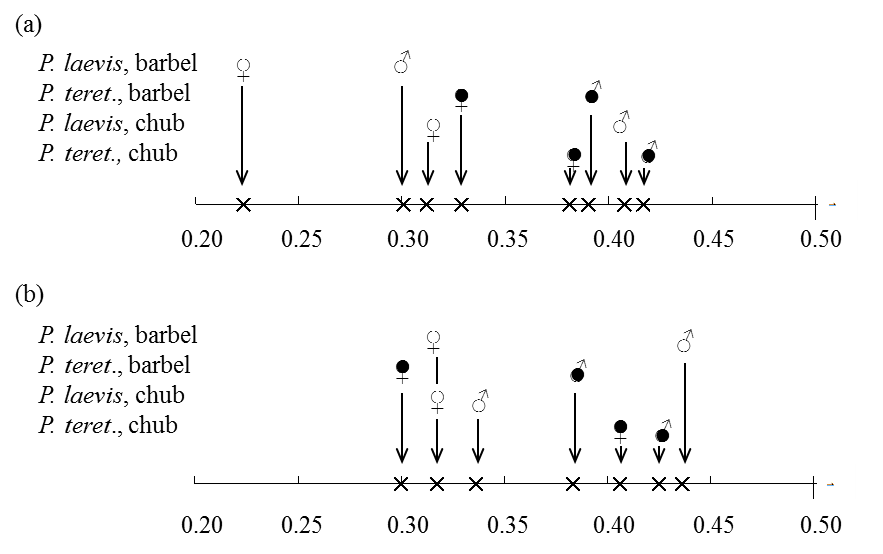


Fig. S2. Distribution of adults of *Pomphorhynchus laevis* and *Pomphorhynchus tereticollis* along the intestinal tract of barbels and chubs from river Ouche (a) and river Vingeanne (b), according to parasite sex. Worm position is given as the mean position in the intestinal tract relative to total intestinal length (from anterior to posterior position). Model comparison using likelihood-ratio test did not show significant effect of predictor variables on the relative position along the digestive tract (arcsin-tranformed). Predictor variables were infection type (heterospecific versus monospecific infection), fish species, parasite species and sex as fixed factors, and individual fish as random factor. Only parasite sex was close to significance threshold, with males occupying a more posterior part (Chi² = 3.41, df = 1, *P* = 0.06)


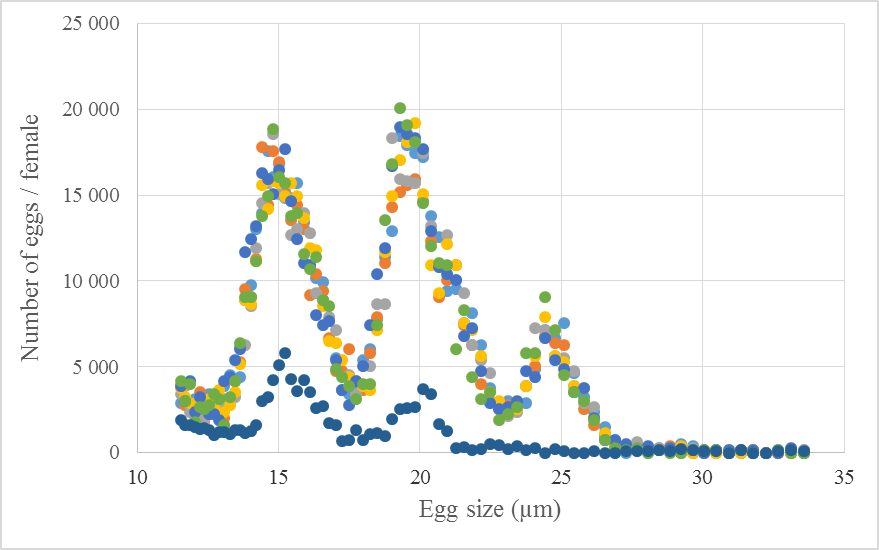


(a) (c)

(b)


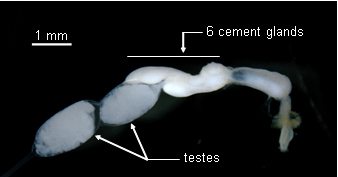


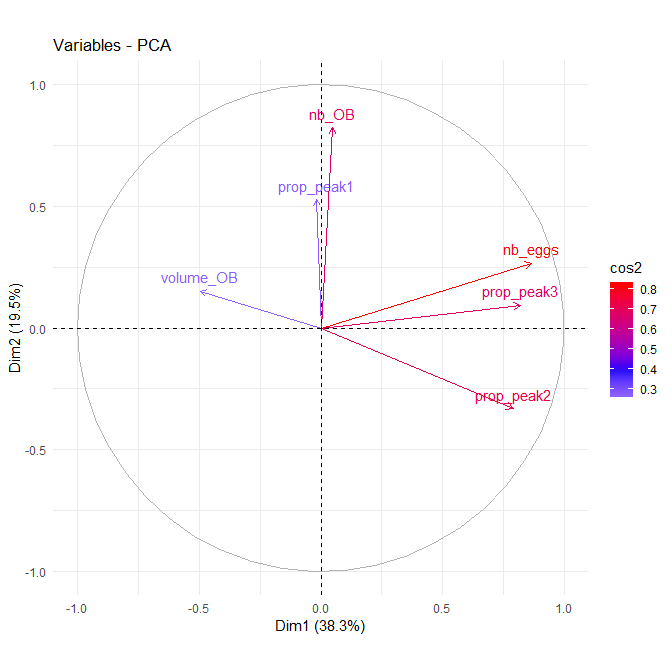


Variable contribution (in %):

PC1 PC2

nb_eggs 32.6 6.1

prop_peak1 0 23.9

prop_peak2 27.2 9.4

prop_peak3 29.2 0.8

nb_OB 0.1 57.9

volume_OB 10.8 2

Fig. S3: Parasite reproductive parameters: (a) Illustration of egg size distribution in egg suspensions from seven individual females, analyzed using particle counter. The three peaks of egg size categories likely correspond to different stages of maturity; (b) Visualization of female reproductive parameters on the PCA factor map: Nb_eggs: total number of eggs per female and prop_peak1, 2, 3: proportion of eggs in each off the three egg size categories; Nb_OB, volume_OB: number and volume of ovarian balls; (c) A photograph of male reproductive tract, showing the pair of testes and cement glands.
